# Expression of concern: Experimental and surface morphological studies of corrosion inhibition on carbon steel in HCl solution using some new hydrazide derivatives

**DOI:** 10.1039/d4ra90028f

**Published:** 2024-03-25

**Authors:** Abd El-Aziz S. Fouda, Samir A. Abd El-Maksoud, Elsherbiny H. El-Sayed, Hazem A. Elbaz, Ashraf S. Abousalem

**Affiliations:** a Chemistry Department, Faculty of Science, Mansoura University Egypt asfouda@hotmail.com +2 050 2202264 +2 050 2365730; b Chemistry Department, Faculty of Science, Port Said University Egypt; c Egyptian Natural Gas Company (GASCO) Egypt; d Quality Control Laboratory, Operations Department, JOTUN Egypt

## Abstract

Expression of concern for ‘Experimental and surface morphological studies of corrosion inhibition on carbon steel in HCl solution using some new hydrazide derivatives’ by Abd El-Aziz S. Fouda *et al.*, *RSC Adv.*, 2021, **11**, 13497–13512, https://doi.org/10.1039/d1ra01405f.

The Royal Society of Chemistry is publishing this expression of concern in order to alert readers that concerns have been raised regarding the reliability of the AFM images in Fig. 10a and b and the IR spectra in Fig. 11 and 13. An investigation is underway, and an expression of concern will continue to be associated with the article until a final outcome is reached.

Laura Fisher

19th March 2024

Executive Editor, *RSC Advances*

## Supplementary Material

